# Integrated Transcriptome and Metabolome Analyses Reveal the Anthocyanin Biosynthesis Pathway in *AmRosea1* Overexpression 84K Poplar

**DOI:** 10.3389/fbioe.2022.911701

**Published:** 2022-06-06

**Authors:** Huiling Yan, Xinxin Zhang, Xiang Li, Xuelai Wang, Hanxi Li, Qiushuang Zhao, Peng Yin, Ruixue Guo, Xiaona Pei, Xiaoqing Hu, Rui Han, Xiyang Zhao

**Affiliations:** College of Forestry and Grassland, Jilin Agricultural University, Changchun, China

**Keywords:** *Populus alba* × *Populus glandulosa* (84K poplar), anthocyanin, AmRosea1, metabolome, transcriptome

## Abstract

*Populus alba* × *Populus glandulosa* (84K poplar) is model material with excellent genetic engineering resource and ornamental value. In our study, *AmRosea1* (*Antirrhinum majus*) was overexpressed in 84K poplar, and the transgenic 84K (AM) poplar with high content of anthocyanin exhibited red pigmentation leaves. The transcriptome analysis between wild type (WT) and AM showed that 170 differentially expressed genes (DEGs) (86 up-regulated and 84 down-regulated) were found, and some DEGs were involved in flavone and flavonol biosynthesis, flavonoid biosynthesis and anthocyanin biosynthesis. The metabolome analysis showed that 13 anthocyanins-related differentially accumulated metabolites (DAMs) were detected in AM. The correlation analysis between DEGs and DAMs were performed, and the results revealed that 18 DEGs, including 11 MYB genes, two *BZ1* genes, one *FG2* gene, one *ANS* gene, and three *IF7MAT* genes, were negatively or positively correlated with 13 DAMs. The phylogenetic analysis demonstrated that there was high homology between *AmRosea1* and *PagMYB113*, and MYB113 co-expressed with BZ1, ANS and DFR directly. Our results elucidated the molecular mechanism of plant color change mediated by anthocyanin biosynthesis pathway, which laid the foundation for the development and utilization of colorful woody plant.

## Introduction

Anthocyanin as natural pigments has abundant physiological and ecological functions, whose content varies greatly depending on the plant variety, season, climate, and plant growth stage (Mobley et al., 2016). Anthocyanin content contributes to the colors such as red, blue and purple of plant vegetative and reproductive organs, thereby determining the application values in landscaping (Quattrocchio et al., 2006; Qiu et al., 2020). Currently, there is growing evidence that anthocyanin has benefits for human health owing to the role as an antioxidant to eliminate free radicals in human body and effectively delaying aging (Chiu et al., 2010; Zhang et al., 2020a). The purple tomato fruit reduces the risks of certain types of cancers and several chronic non-communicable diseases (CNCDs), such as diabetes, hypertension and obesity (Raiola et al., 2014). Anthocyanin biosynthesis is a specific branch of the phenylpropanoid pathway, which is one of the comprehensively studied secondary metabolic pathway (Zong et al., 2019; Yi et al., 2020). The pathway of anthocyanin biosynthesis involves many enzymes, whose coding genes are mainly divided into two classes, one consists of early biosynthetic genes (EBGs, such as *CHS*, *CHI*, and *F3H*), and the other consists of late biosynthetic genes [LBGs, such as *DFR*, *ANS*, and *BZ1* (encodes UDP glucose flavonoid glucosyl-transferase, UFGT)] (Diao et al., 2004; Zong et al., 2019; Yan et al., 2021).

Studies on numerous species have explored key genes or transcription factors (TFs) that regulate anthocyanin biosynthesis. For example, MYELOBLASTOSIS (MYB) TFs, regulators of plant metabolic network, are used as activators alone or combining with other TFs to form the myeloblastosis-basic helix-loop-helix (bHLH)-tryptophan-aspartate (WD40) (MBW) complex that regulates the expression of anthocyanin biosynthesis structural genes (Xie et al., 2012; Xu et al., 2020). Multiple comparisons of anthocyanin-related genes reveal that the MYB-R3 domain contains a conserved bHLH binding motif (D/E)Lx2 (R/K)x3Lx6Lx3R, and R2R3-MYB usually forming a complex, thereby activating the expression of structural genes and promoting the accumulation of anthocyanin (Albert, et al., 2011; Cao, et al., 2017). In *Paeonia suffruticosa*, PsMYB12 interacts with bHLH and WD40 to form a protein complex that directly activate the expression of *PsCHS*. The activation leads to the pigmentation thereby exhibiting blotch, while the loss of the *CHS* activity contributes to albino flower (Gu, et al., 2019). In *Anthurium andraeanum*, *AaMYB2* is considered to be a potential target that controls corolla color and involves in the joint expression of *AaCHS*, *AaF3H* and *AaANS* (Li, et al., 2016). In other plants, overexpression of *MYB118* or *MYB119* in poplar may change leaf color (Cho et al., 2016; Wang et al., 2019a). *AmRosea1* belonging to MYB family is isolated from snapdragon (*A. majus*), and the overexpression of this gene is able to affect anthocyanin biosynthesis in snapdragons and tobacco (Schwinn. 2006; Zhang et al., 2013). Meanwhile, Rosea1 can interact with Delia to increase anthocyanin accumulation, which turns orange carrots into purple (Schwinn. 2006; Sharma et al., 2020).

*Populus alba* x *P.* glandulosa (84K poplar) is a fast-growing hybrid which derives from a breeding program in South Korea, and it is introduced into China until 1984 (Qiu et al., 2019; Huang et al., 2021). Scientifically, 84K poplar is a model material for studying on growth and development processes of perennial trees due to extensive genetic resources and perfect genetic transformation methods (Feng et al., 2015; Huang et al., 2021). In this study, *AmRosa1* was transferred into the 84K male poplar to obtain *AmRosea1* overexpression transgenic 84K poplar. The phenotype of *AmRosea1-*overexpressing plants was charactered, while gene expression profile and metabolites associated with anthocyanin biosynthesis or anthocyanin accumulation were analyzed. The results would enhance the understanding of other significant structural genes in anthocyanin biosynthesis pathway regulated by *AmRosa1*, which laid the foundation for the development and utilization of colorful woody plants. Furthermore, the preference that 84K male poplar were selected as transgenic receptors for changing plants color could reduce the pollution of poplar catkins in spring, which could optimize the biological safety of poplar as street trees.

## Materials and Methods

### Plant Materials

*Populus alba* × *P. glandulosa* (84K poplar) were obtained from College of Forestry and Grassland, Jilin Agricultural University (Changchun, China). Wild type 84K poplar (named WT) and the highest *AmRosea1* overexpression transgenic 84K poplar L-24 (named AM) were cultured from January to June for further analyses (Supplementary Figure S1). The functional leaves (the third to fifth leaves from the main branches) of WT and AM without mechanical damage or disease were mixed respectively for performing transcriptional and metabolic analyses with three biological replicates (named WT-1, WT-2, WT-3 and AM-1, AM-2, AM-3). And storing at −80°C until transcriptional and metabolic analyses were performed. They performed for transcriptional and metabolic analyses.

### Vector Construction and Genetic Transformation

The *AmRosea1* gene fragment sequence was identified from NCBI database (GenBank: DQ275529, *A. majus*). The 663 bp gene fragment was synthesized into PUC57 vector (Beijing Genomics Institution, Beijing, China). After confirming sequence, *AmRosea1* gene (amplification primers were F: 5′-ATG​GAA​AAG​AAT​TGT​CGT​GGA​G-3′, R: 5′- TTA​ATT​TCC​AAT​TTG​TTG​GGC​CT-3′) was cloned and inserted into pROKII vector according to Liu et al. (2020). The vector was transferred into 84 K poplar using the *Agrobacterium tumefaciens*-mediated leaf disc transformation method (Cho et al., 2016). Transgenic shoots were cultured on MS medium with 20 mg/L kanamycin for kanamycin-resistant buds, and wild type line was cultured on MS medium. The genome DNA and total RNA were extracted for PCR using amplification primers and RT-qPCR (the primers were F: 5′-AGA​GTA​TGG​TGA​AGG​GAA​ATG​G-3′, R: 5′-CCG​ACC​TCT​TTT​GAT​ATT​TGG​C-3′).

### Observation of Stem Cross Section

Cross sections of poplar stems were prepared by hand-cutting, and the anthocyanin pigment accumulation without staining of one-month-old WT and AM was observed (Cho et al., 2016).

### The Detection of Total Anthocyanin Content

The contents of total anthocyanin were detected according to Wang et al. (2019a) using functional leaves (the third to fifth leaves from the main branches) of six-month-old WT and AM.

### RNA-Seq and RT-qPCR Verification

The total RNA extraction and library construction were carried out according to Li et al. (2021). The libraries were sequenced on the Illumina HiSeq platform with an average reading length of 125 bp/150 bp. As for RT-qPCR verification, the total RNA from each sample was extracted using RNA extraction kit (Tiangen, Beijing, China) according to the manufacturer’s instruction. The cDNA was synthesised using the PrimeScript RT reagent Kit with gDNA Eraser (TaKaRa, Kyoto, Japan). The primers for RT-qPCR were designed using the Integrated DNA Technologies (https://sg.idtdna.com/pages), and the sequences were listed on Supplementary Table S1. *PtActin* (amplification primers were F: 5′-AAT​ACC​CCA​TTG​AGC​ACG​G-3′, R: 5′- ACT​CAC​ACC​ATC​ACC​AGA​ATC-3′) were selected as an internal reference gene. The amplification system and procedure were carried out according to Li et al. (2021). The relative expression level of genes was calculated using the 2^−∆∆CT^ method (Livak and Schmittgen. 2001).

### Transcriptome Analysis

The 84K poplar genome was downloaded and used for the reference genome (Qiu et al., 2019; Huang et al., 2021). Spliced Transcripts Alignments to a Reference (STAR) (Dobin et al., 2014) was used as aligner to map the sequencing reads to the reference genome with parameters recommended by RSEM. The Fragments Per Kilobase of transcript per Million fragments mapped (FPKM) of each gene were calculated based on the gene length. The functions of unigenes were annotated by the NCBI non-redundant protein sequences (Nr), euKaryotic Ortholog Groups (KOG), a manually annotated and curated protein sequence database (Swiss-Prot), Gene Ontology (GO), and Kyoto Encyclopedia of Genes and Genomes (KEGG) databases (Li et al., 2021). The analysis of differential expression genes (DEGs) between two-sample comparison was performed using the DESeq2 package (1.20.0) with *p-value* < 0.05 and |log2 foldchange| ≥ 1. The resulting *p* values were adjusted using the Benjamini and Hochberg’s false discovery rate (FDR). The analyses of GO and KEGG were performed according to Li et al. (2021) and Liu et al. (2021). Structural genes related to the anthocyanin biosynthesis pathway were screened from DEGs.

### Metabolite Extraction and Metabolite Profiling

The prepared leaf samples were used to extract metabolite according to Zhang et al. (2021). Normalized data of each sample were used to analyzing differentially accumulated metabolites (DAMs). The detailed procedure for metabolite analysis was performed as previously described by Li et al*.* (2021).

### Correlation Analysis of Transcriptome and Metabolome

Pearson correlations were calculated for the integrative analysis between the various DAMs and DEGs related to anthocyanin biosynthesis. The coefficient (*r*) value >0.9 or < −0.9 represented crucial relationships between metabolome and transcriptome. Correlation Network was constructed using the OmicStudio tools (https://www.omicstudio.cn/tool).

### Phylogenetic Tree Analysis and Co-Expression Network Construction

Sequences were obtained from transcriptome for multiple sequence alignment, which was performed by BioEdit. The phylogenetic tree was constructed based on 12 protein sequences using MEGA ver. 7.0. The co-expression network of MYB and other structural genes were constructed by string (https://cn.string-db.org/) using sequences in *Arabidopsis thaliana* to identify the relationship between MYB and structural genes.

### Statistical Analysis of Data

Data analysis were performed using Excel 2021, and SPSS version 26.0 (International Business Machines, NY, United States). Data indicated mean ± SD with three biological replications. Asterisks indicated significant differences between two lines based on student’s *t*-test (**p < 0.01).

## Results

### Generation and Characterization of *AmRosea1* Transgenic 84K Poplar

**FIGURE 1:**
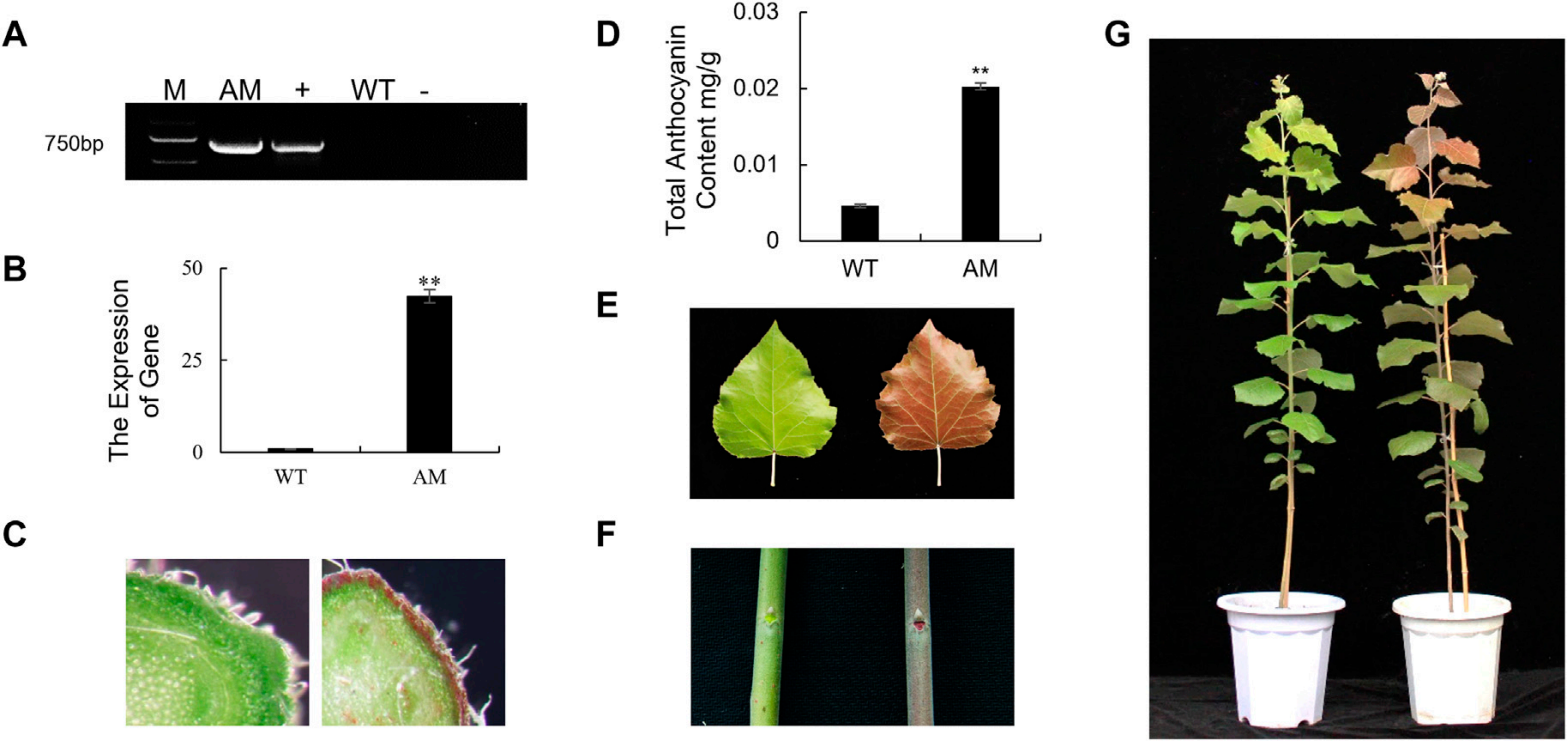
Generation and characterization of *AmRosea1* transgenic 84K poplar. The whole picture on the left is wild-type (WT) 84K poplar, and AM on the right is *AmRosea1* transgenic material. **(A)**. Total DNA was extracted from leaf tissue for PCR verification. M, 2000 bp DNA Marker; AM, *AmRosea1* transgenic poplar 84K; +, positive control; WT, wild type poplar 84K; -, negative control. **(B)**. The expression of *AmRosea1* in WT and AM. The stem cross cutting of one-month-old WT and AM **(C)**. **(D)** The total anthocyanin contents of six-month-old WT and AM functional leaves. The phenotypic observation of functional leaves **(E)**, stems **(F)**, and the whole plants **(G)** of six-month-old WT and AM. Error bars represent the SDs from three biological replicates. Statistical differences were determined by a student’s *t*-test (**p < 0.01).

To understand the specific functions of *AmRosea1* related to anthocyanin biosynthesis in poplar, *AmRosea1* was overexpressed in 84K poplar*.* The exogenous *AmRosea1* gene was detected by PCR and RT-qPCR. Amplified bands about 700 bp were found in positive plasmid (35S: *AmRosea1*) and AM line, which were not found in negative control and WT (Figure 1A). Meanwhile, the expression of *AmRosea1* was extremely significantly up-regulated in AM line (*p* < 0.01) (Figure 1B). The phenotype of WT and AM was characterized. Compared to WT, red pigmented cells were found in the AM cortex from cross sections of one-month-old plants (Figure 1C). The total anthocyanin content in functional leaves of six-month-old AM was over two times that of WT (Figure 1D). The leaves, stems, and whole plant of six-month-old AM exhibited deeply red pigment, while none pigmentation was found in WT (Figures 1E–G). The total anthocyanin content in functional leaves of six-month-old AM was over two times that of WT (Figure 1F). These results showed that AM was generated and confirmed. The AM line exhibited red pigmentation during the growth stage, which were associated with high anthocyanin content.

### Sequencing Quality Evaluation of Transcriptome and RT-qPCR Verification

**FIGURE 2:**
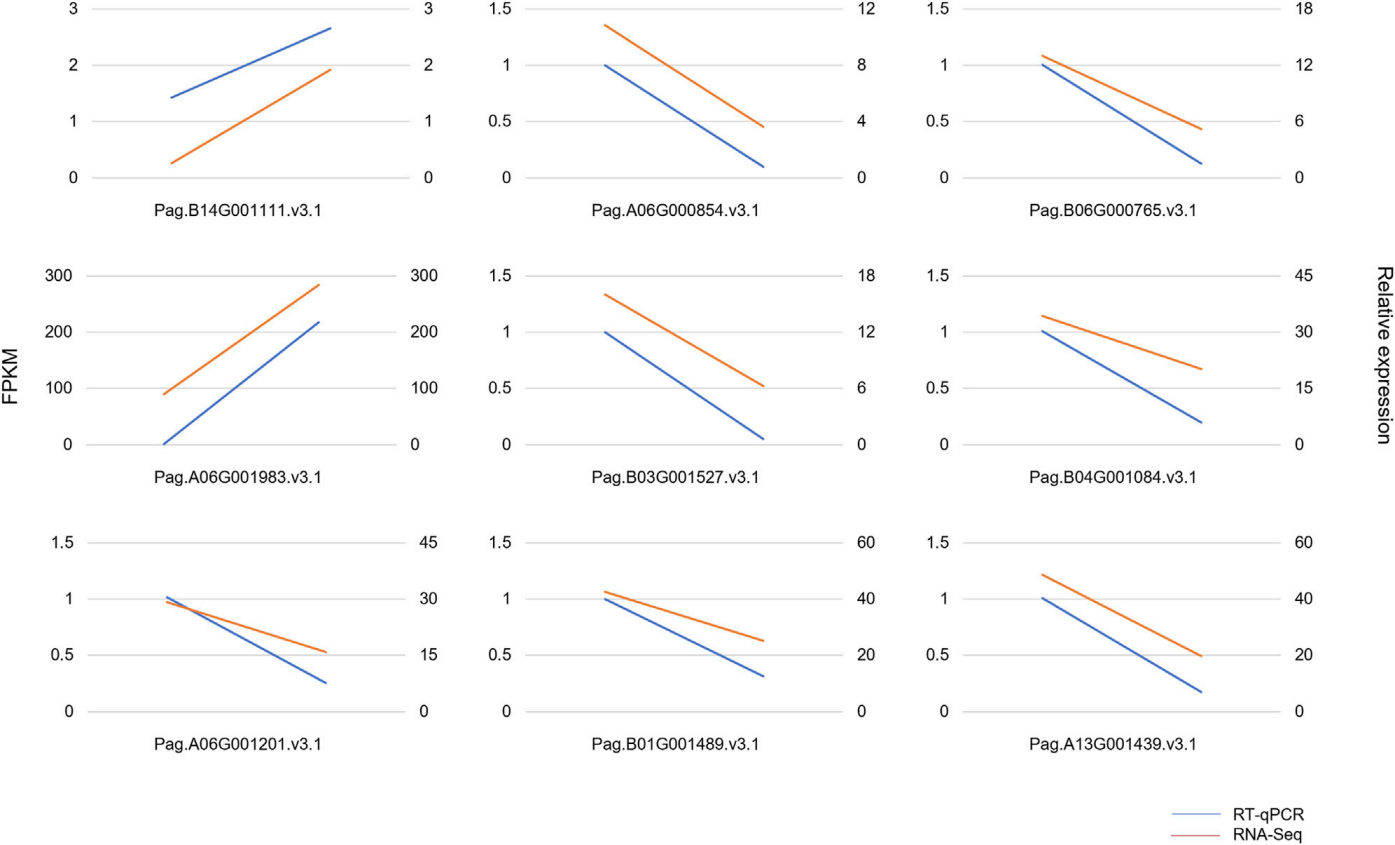
Quantitative real-time PCR verification of RNA-Seq. The red lines represent the results of RT-qPCR, and the blue lines represent the results of RNA-Seq. The *y*-axis on the left represents the FPKM value obtained by RNA-Seq. The *y*-axis on the right shows the gene relative expression levels analyzed by RT-qPCR. The endpoints on the left represent gene expression in WT, and endpoints on the right represent gene expression in AM.

To analyze the gene expression profiles, RNA-Seq was performed using six-month-old WT and AM leaves. As a result, a total of 428,174,950 raw sequencing reads were generated from six samples, a total of 415,152,012 clean reads were finally obtained after removing low-quality data. The alignment of clean reads in six samples against the reference genome was from 95.06 to 97.19%, the Q30 percentages were all over 91.1% (Table 1). To test the reliability of RNA-Seq, nine genes were selected for verification, using RT-qPCR. The gene expression detected by RT-qPCR showed a similar pattern to that detected by the RNA-Seq (Figure 2). These results proved the accuracy of the transcriptome so that the transcriptome could be used for further analysis.

**TABLE 1:** The sequencing quality evaluation of RNA-Seq.

Sample	Raw reads	Clean reads	Clean bases (Gb)	Reads mapped	Q30 (%)	Unique mapped
AM-1	77,404,290	74,976,426	11.25	71,322,542 (95.13%)	91.28	61,463,705 (81.98%)
AM-2	72,578,404	70,146,494	10.52	66,786,465 (95.21%)	91.14	57,497,415 (81.97%)
AM-3	68,462,758	66,282,242	9.94	64,418,579 (97.19%)	92.41	55,671,528 (83.99%)
WT-1	68,278,232	66,369,748	9.96	63,023,334 (94.96%)	91.10	54,624,476 (82.30%)
WT-2	77,381,546	74,965,850	11.24	71,260,587 (95.06%)	91.37	61,545,330 (82.10%)
WT-3	64,069,720	62,411,252	9.36	59,325,283 (95.06%)	91.12	51,369,763 (82.31%)

### Transcriptome Analysis of *AmRosea1* Overexpression in 84K Poplar

**FIGURE 3:**
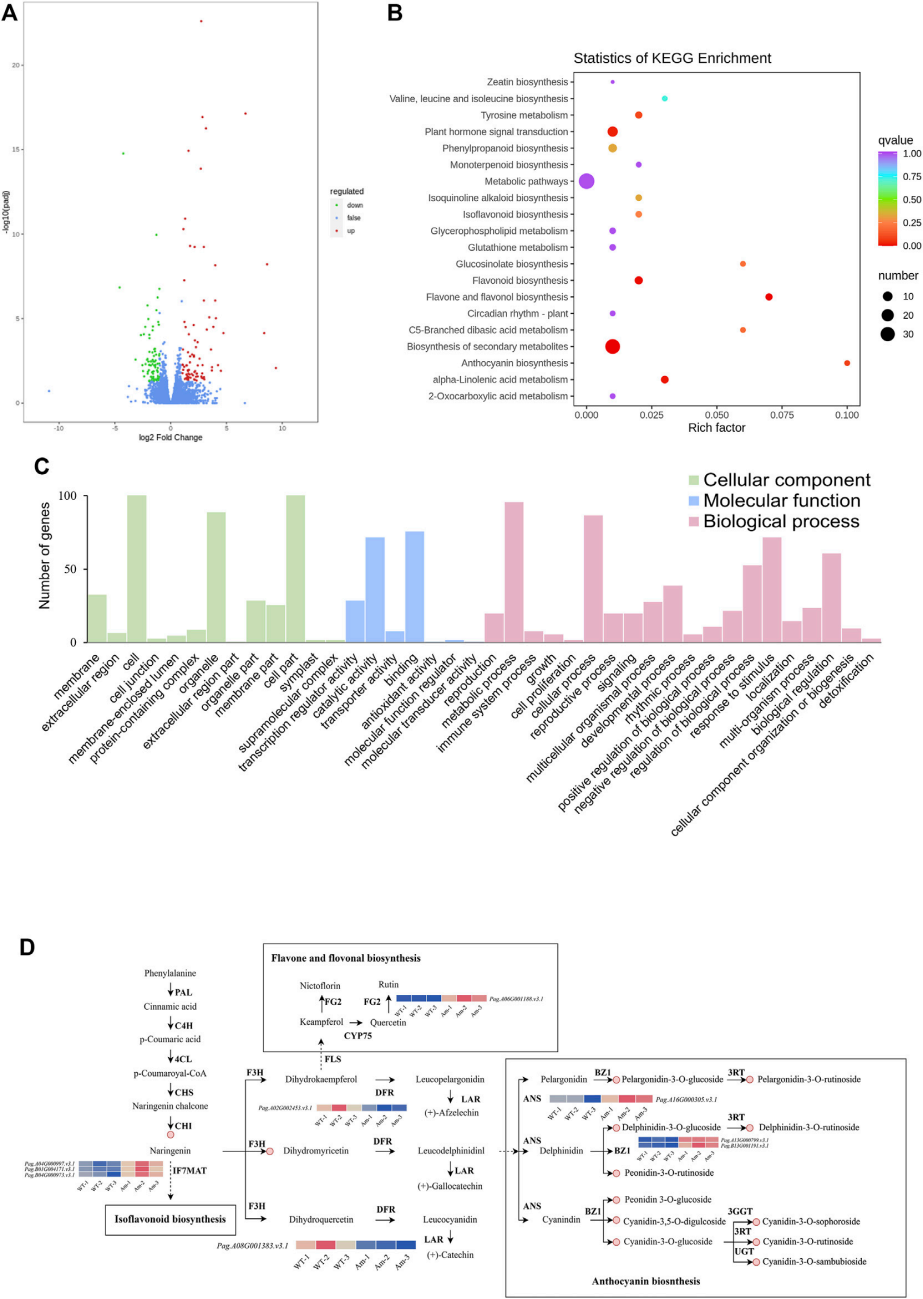
Identification of the differentially expressed genes (DEGs) in WT vs. AM. **(A)**. Numbers of DEGs in volcano plots. **(B)**. KEGG enrichment analysis of the DEGs. **(C)**. Gene ontology (GO) classification of DEGs. **(D)**. Differential expression of genes in flavonoid and anthocyanin biosynthesis pathways in poplar 84K leaf (WT vs. AM). PAL, phenylalanine ammonia-lyase; C4H, cinnamic acid 4-hydroxylase; 4CL, 4-coumarate CoA ligase; CHS, chalcone isomerase; CHI, chalcone isomerase; FG2, flavonol-3-O-glucoside l-rhamnosyltransferase; CYP75, flavonoid 3′-monooxygenase; F3H, lavanone 3-hydroxylase; FLS, flavonol synthase; IF7MAT, isoflavone 7-O-glucoside-6″-O-malonyltransferase; DFR, dihydroflavonol 4-reductase; LAR, leucoanthocyanidin reductase; ANS, anthocyanidin synthase; BZ1, anthocyanidin 3-O-glucosyltransferase; 3RT, pelargonidin 3-O-rutinoside + UDP; 3GGT, anthocyanidin 3-O-glucoside 2″-O-glucosyltransferase; UGT, anthocyanidin 3-O-glucoside 5-O-glucosyltransferase.

Based on the transcriptome, 170 genes (86 up-regulated and 84 down-regulated) were found to be differentially expressed in AM compared with WT (Figure 3A). KEGG analysis demonstrated that DEGs were mainly enriched in biosynthesis of secondary metabolites, flavonoid biosynthesis and anthocyanin biosynthesis, indicating that *AmRosea1* was related to anthocyanin biosynthesis (Figure 3B). DEGs were assigned to the GO categories, according to the biological process, cellular component, and molecular function (Figure 3C; Supplementary Table S2). In the biological process, 20, 38, and eight DEGs were involved in anthocyanin biosynthesis or metabolic pathway, flavonoid biosynthesis or metabolic process, and flavone biosynthesis or metabolic pathway, individually (Supplementary Figure S2). For the molecular function, three DEGs connected with anthocyanidin 3-O-glucosyltransferase activity were found. Additionally, nine DEGs encoding five enzymes, including *PagDFR* (*Pag.A02G002453. v3.1*) and *PagLAR* (*Pag.A06G001383. v3.1*), *PagFG2* (*Pag.A06G001188. v3.1*), *PagIF7MAT* (*Pag.B01G004171. v3.1*, *Pag. B01G000997. v3.1, and Pag. B04G000973. v3.1*), *PagANS* (*Pag.A16G000305. v3.1*), *PagBZ1* (*Pag.A13G000799. v3.1* and *Pag. B03G001191. v3.1*), were identified, which participated in flavone and flavonol biosynthesis pathway (ko00944), isoflavonoid biosynthesis pathway (ko00943) and anthocyanin biosynthesis pathway (ko00942) (Supplementary Table S3). Compared to WT, *PagDFR* were down-regulated in AM, whereas other LBGs, *PagANS*, *PagBZ, PagFG2* and *PagIF7MAT* were also up-regulated in AM (Figure 3D). These results suggested that the overexpression of *AmRosea1* in 84K poplar might affect secondary metabolite biosynthesis and metabolism by regulating gene expression, especially the flavone and flavonol biosynthesis, flavonoid biosynthesis and anthocyanin biosynthesis.

### Metabolome Analysis of *AmRosea1* Overexpression Transgenic 84K Poplar

**FIGURE 4:**
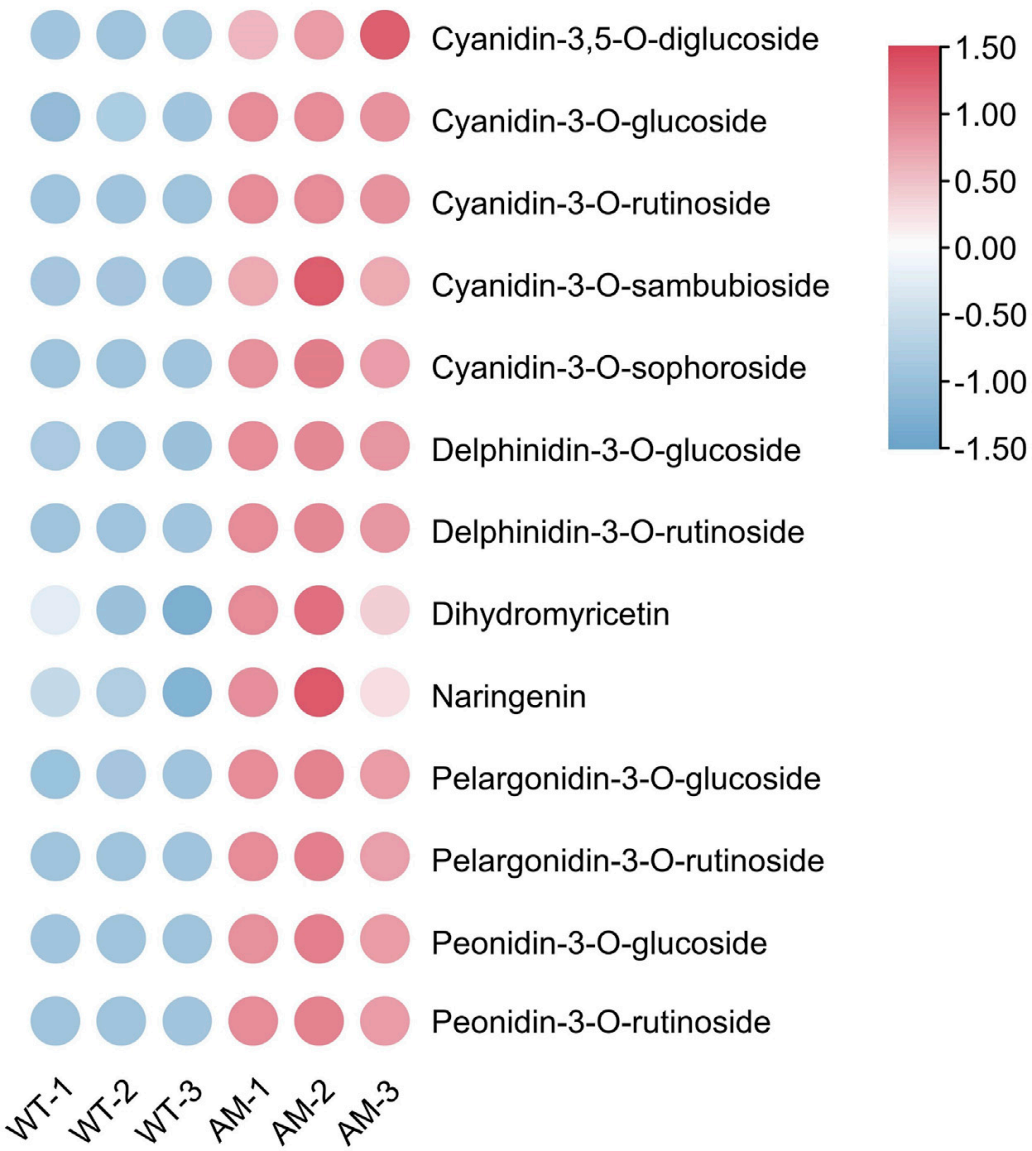
The differentially accumulated metabolites (DAMs) of anthocyanin accumulation between WT vs. AM.

The differentially accumulated metabolites (DAMs) were explored using metabolome. The levels of 13 anthocyanins metabolites increased, including five cyanidin, two delphinidin, two flavonoid, two pelargonidin, and two peonidin. They were cyanidin-3,5-O-diglucoside, cyanidin-3-O-glucoside, cyanidin-3-O-rutinoside, cyanidin-3-O-sambubioside, cyanidin-3-O-sophoroside, delphinidin-3-O-glucoside, delphinidin-3-O-rutinoside, pelargonidin-3-O-glucoside, pelargonidin-3-O-rutinoside, peonidin-3-O-glucoside, peonidin-3-O-rutinoside, dihydromyricetin, and naringenin. Among these metabolites, the levels of cyanidin-3-O-glucoside, delphinidin-3-O-glucoside, and delphinidin-3-O-rutinoside in AM line were significantly higher than WT (Figure 4; Supplementary Table S4). The results proposed that the overexpression of AmRosea1 in 84K poplar increased the level of anthocyanins-related metabolites, especially cyanidin-3-O-glucoside, delphinidin-3-O-glucoside and delphinidin-3-O-rutinoside, thereby controlling anthocyanin biosynthesis.

### Correlation Analysis Between DEGs and DAMs

**FIGURE 5:**
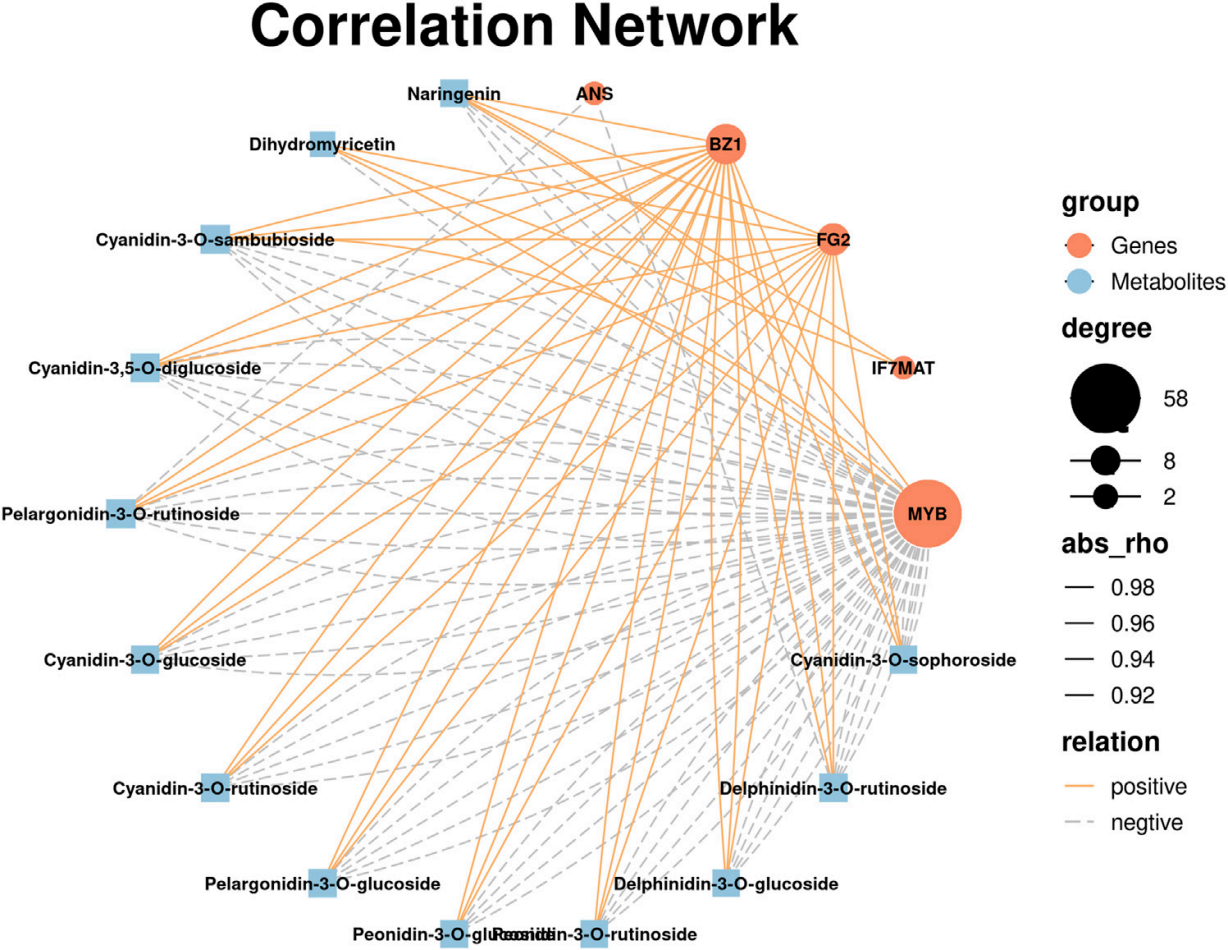
The construction of regulatory network between DEGs and DAMs. rho represents the Pearson correlation coefficient (*r*). Degree represents the gene number. Relation represents the correlations with a coefficient (*r*) value >0.9 (positive) or < −0.9 (negative).

To understand the relationship between DEGs and DAMs in anthocyanin biosynthesis, the integrative analysis was performed. On the basis of the correlation analysis between DEGs and DAMs with coefficients of *r* > 0.9 or < -0.9, *ANS* gene was significantly negatively associated with pelargonidin-3-O-rutinoside and delphinidin-3-O-rutinoside, and *IF7MAT* genes were positively associated with dihydromyricetin and naringenin. *BZ1* genes were significantly positively related to cyanidin-3,5-O-diglucoside, cyanidin-3-O-glucoside, cyanidin-3-O-rutinoside, cyanidin-3-O-sambubioside, cyanidin-3-O-sophoroside, delphinidin-3-O-glucoside, delphinidin-3-O-rutinoside, naringenin, pelargonidin-3-O-glucoside, pelargonidin-3-O-rutinoside, peonidin-3-O-glucoside, and peonidin-3-O-rutinoside. *FG2* genes were significantly positively involved in cyanidin-3,5-O-diglucoside, cyanidin-3-O-glucoside, cyanidin-3-O-rutinoside, cyanidin-3-O-sophoroside, cyanidin-3-O-sambubioside, delphinidin-3-O-glucoside, delphinidin-3-O-rutinoside, dihydromyricetin, naringenin, pelargonidin-3-O-glucoside, pelargonidin-3-O-rutinoside, peonidin-3-O-glucoside, and peonidin-3-O-rutinoside. *MYB* genes were only positively correlated with cyanidin-3-O-sambubioside, naringenin, dihydromyricetin (Supplementary Table S5). The regulatory network where five structural genes regulated 13 mentioned metabolites positively or negatively were constructed (Figure 5). These results showed that the contents of anthocyanin mainly affinitive with MYB genes and other structural genes, *FG2* genes, *BZI* genes, *ANS* genes and *IF7MAT* genes as such in AM.

### Co-Expression Network of *AmRosea1* in 84K Poplar

**FIGURE 6:**
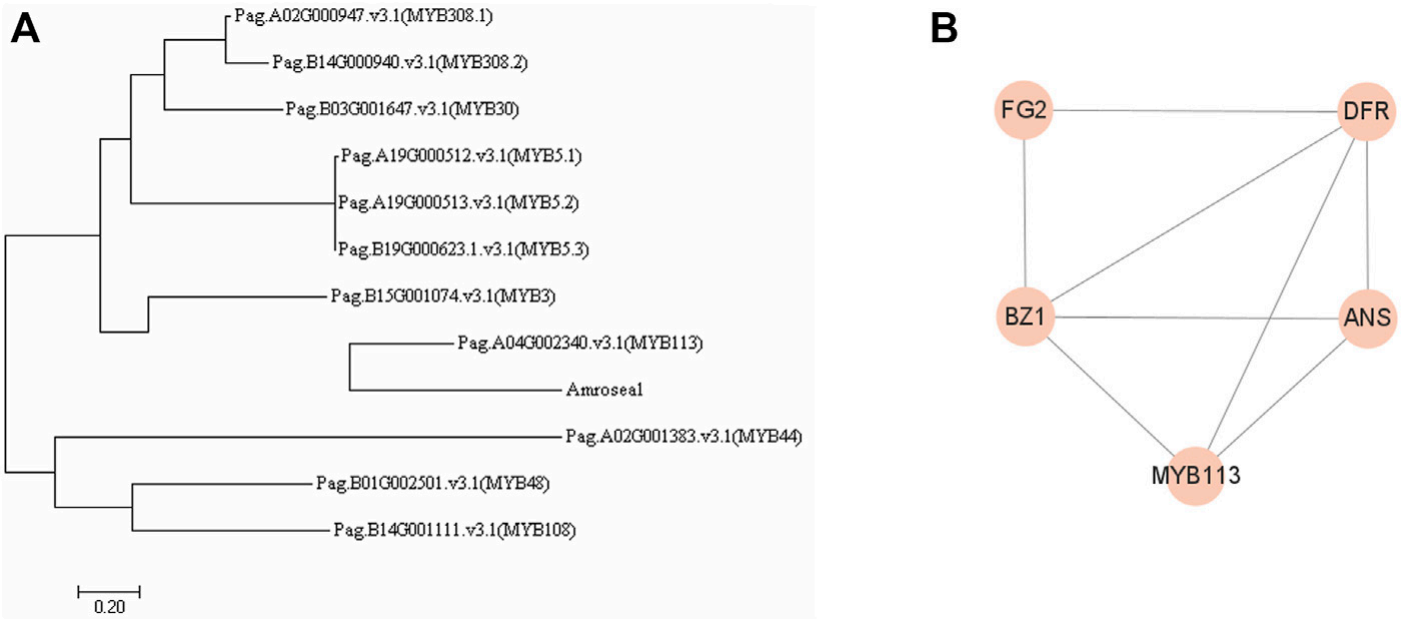
Phylogenetic tree analysis and construction of co-expression network. **(A)**. The phylogenetic tree between MYBs and AmRosea1 using MEGA seven software. **(B)**. The co-expression network of MYB113 used String (https://cn.string-db.org/).

In order to explore the mechanism of *AmRosea1*, we conducted the phylogenetic analysis between *AmRosea1* and DEGs belonging to MYB in poplar 84K. The phylogenetic analysis revealed that there was high homology between *AmRosea1* and *PagMYB113* (*Pag.A04G002340. v3.1*) (Figure 6A; Supplementary Table S6). Therefore, MYB113 was selected to construct co-expression network in *A. thaliana* for enhancing the understanding of regulatory relationship, which showed that *MYB113* co-expressed with *BZ1*, *ANS* and *DFR* directly (Figure 6B). The results suggested that *AmRosea1* might co-express with *BZ1*, *ANS* and *DFR* directly, thereby affecting anthocyanin biosynthesis in AM.

## Discussion

*Populus alba* x *P.* glandulosa originally derived from South Korea, which is one of the major tree species cultivated widely in China recently. 84K poplar is used as a model plant for understanding molecular mechanisms and economic objectives of woody plants. In previous studies, the researches of 84K poplar focused on gene expression patterns under nitrogen deficiency stress (Liu et al., 2021), cavitation and frost fatigue (Feng et al., 2015), molecular mechanisms of wood formation (Xiao et al., 2021), whereas fewer studies that exogenous gene caused the leaves to turn red in 84K poplar were reported. The conjoint analysis of transcriptome and metabolome was a potential method to explore some specific molecular mechanisms based on gene expression patterns and metabolite profiles. This method had been used in many species, for example, the anthocyanin and proanthocyanin accumulation were analyzed by transcriptional and metabolic analysis in red mutant pear (Zhang et al., 2020b), the candidate genes involved in anthocyanin accumulation of green and red leaves in *Acer* at the growth stage were explored (Zhang et al., 2021), the metabolite accumulation and gene expression patterns in inner and outer seed coats of hard-seeded and soft-seeded pomegranate were detected (Qin et al., 2020). In our study, transcriptome and metabolome of AM exhibiting red pigmentation in leaves, stems and whole plants were integrated to elucidate the molecular mechanism of plant color change.

### The Anthocyanin Metabolites Have Effect on Plant Pigment

The various anthocyanins make plants exhibit different colors, six categories of metabolites, including malvidin, pelargonidin, cyanidin, peonidin, delphinidin and leucocyanidin, participate in anthocyanin biosynthesis (Yan et al., 2021). Red phenotype was closely associated with the types and contents of red pigment (Li et al., 2021). The results of Cho et al. (2016) showed that the overexpression of *PtrMYB119* affected the accumulation of cyanidin-3-O-glucoside, thereby promoting anthocyanin biosynthesis, which changed leaf color. And the same result was found in the peel of red longan (Yi et al., 2021). Zhang et al. (2021) found that cyanidin 3-O-glucoside, pelargonidin 3-O-glucoside and delphinidin 3-O-glucoside were significantly increased in red-leaf acer. In this study, the AM with high anthocyanin content was generated and confirmed, which exhibited red pigmentation in leaves, stems and whole plant during the growth stage (Figures 1C–E–G). The metabolome analysis showed that 13 anthocyanins-related metabolites increased in transgenic 84K poplar, especially cyanidin-3-O-glucoside improved greatly (Figure 4, Supplementary Table S4), which was consistent with other studies (Cho et al., 2016; Zhang et al., 2021). These results proposed that the levels of anthocyanin-related metabolites, cyanidin-3-O-glucoside as such, affected anthocyanin content, thereby changing plant color.

### Integated Analysis Between DEGs and DAMs

The accumulation of anthocyanin is mainly affected by structural genes and TFs (Wang et al., 2019b). Structural genes control the metabolite content in anthocyanin biosynthesis pathway by changing the expression levels of upstream and downstream genes (Li et al., 2019). Transcription factors can influence structural genes to indirectly regulate anthocyanin metabolite accumulation (Zhang et al., 2021) On the basis of correlation network, *DFR* genes down-regulated in AM were not related to 13 mentioned metabolites (Figure 3D, Figure 5). *BZ1*, *FG2*, and *IF7MAT* genes were positively associated with anthocyanin-related metabolites, whereas *ANS* genes were negatively associated with anthocyanin-related metabolites. Among these four kinds of genes, *FG2* and *IF7MAT* genes up-regulated in AM were involved in flavone biosynthesis and isoflavonid biosynthesis pathways, which determined the increases of two and 13 anthocyanin-related metabolites, respectively. *BZ1* genes up-regulated in AM participated in anthocyanin biosynthesis directly, which also led the accumulation of 13 anthocyanin-related metabolites. Cyanidin-3-O-glucoside with highest level in AM was positively related to *FG2* and *BZ1* genes (Figure 5; Supplementary Table S5; Figure 3D). These results suggested that the up-regulated *IF7MAT* genes involved in isoflavonid biosynthesis might promote anthocyanin biosynthesis pathway. Up-regulated *BZ1* genes facilitated the synthesis of intermediate products, cyanidin-3-O-glucoside, delphinidin-3-O-glucoside, and pelargonidin-3-O-glucoside as such, followed by improving the levels of end-products, cyanidin-3-O-rutinoside, cyanidin-3-O-sambubioside, cyanidin-3-O-sophoroside, delphinidin-3-O-rutinoside, pelargonidin-3-O-rutinoside, and peonidin-3-O-rutinoside as such. These changes of DEGs and DAMs were major in the increased anthocyanin content.

### The Functions of *AmRosea1* in Anthocyanin Biosynthesis

Except for mentioned structural genes, MYB TFs used as activators alone or forming ternary-complexes with bHLH and WD40 regulate anthocyanin biosynthesis (Xie et al., 2012; Xu et al., 2020). *MrMYB1* (*Myricarubra Chinese*) activated the promoter of *NtDFR* depending on the existence of bHLH, and the co-expression of *MrMYB1* with the bHLH genes would increase the anthocyanin (Niu, et al., 2010). *AmRosea1* as the member of MYB TFs could activate anthocyanin biosynthesis by over-expression in herbaceous plants (Zhang et al., 2013). For example, Rosea1 interacted with Delia to enhance anthocyanin in carrot and *Salvia miltiorrhiza*, thereby changing plant color (Wang et al., 2013; Sharma et al., 2020). In this study, the anthocyanin content and anthocyanin-related metabolites increased in AM, which demonstrated that the anthocyanin biosynthesis pathway was activated by AM. Most of MYB genes were negatively correlated with anthocyanin-related metabolites except for cyanidin-3-O-sambubioside, dihydromyricetin and naringenin. Additionally, none of genes related to bHLH were identified on the basis of transcriptome, these results proposed that *AmRosea1* deriving from snapdragon as an exogenous gene regulated anthocyanin biosynthesis excluding forming classical ternary-complex. In this study, the overexpression of *AmRosea1* activated the anthocyanin biosynthesis pathway, and the expression of some structural genes significantly changed, including *DFR*, *ANS*, *FG2*, *IF7MAT*, *BZ1*, *LAR* (Supplementary Figure S3). The *MdMYB1*, *MdDFR* and *MdUFGT* (encoded by *BZ1*) in apple (*Malus domestica*) could activate the anthocyanin synthesis pathway (Takos, et al., 2006). The heterologous *MYB1* of *Muscari botryoides* Mill. was inserted in tobacco could independently regulate *DFR*, and the purple anthocyanin pigment was found in transgenic tobacco leaves, petals, anthers and calyx. (Chen, et al., 2019). *FhMYB5* of *Freesia hybrida* was inserted in tobacco, which specially recognized *NtLAR* promoter site to promote the anthocyanin accumulation (Li, et al., 2018). These results show that MYB genes activate the anthocyanin pathway by binding to structural genes. The phylogenetic analysis showed that there was high homology between *AmRosea1* and *PagMYB113* (Pag.A04G002340. v3.1) (Figure 6A, Supplementary Table S6). The co-expression network showed that MYB113 might interact with BZ1, ANS and DFR directly in *A. thaliana* (Figure 6B). Therefore, we inferred that A*mRosea1* might play the similar role with *PagMYB113.* The overexpression of this gene might activate the expression of *ANS* and *BZ1* genes directly, thereby improving the contents of anthocyanin and anthocyanin-related metabolites, which turned green 84K poplar plants into red. Our results enhanced the understand of anthocyanin biosynthesis pathway to plant color change, which provided references for the development and utilization of colorful woody plant.

## Conclusion

In summary, the *AmRosea1* overexpression transgenic 84K poplar with high total anthocyanin content exhibited red pigmentation in leaves, stems and whole plant. Genes involved in flavone and flavonol biosynthesis, flavonoid biosynthesis and anthocyanin biosynthesis expressed differentially, while 13 anthocyanins-related metabolites that had affinity with *MYB*, *ANS*, *FG2*, *BZ1*, and *IF7MAT* genes increased, especially the levels of cyanidin-3-O-glucoside, delphinidin-3-O-glucoside and delphinidin-3-O-rutinoside improved greatly. *PagMYB113* that had high homology with *AmRosea1* might be co-expressed with *BZ1*, *ANS* and *DFR* directly in 84K poplar. Overall, all results might suggest that the overexpression of *AmRosea1* in 84K poplar increased the contents of anthocyanin and anthocyanins-related metabolites through regulating *BZ1*, *ANS* and *DFR* directly, thereby turning green 84K poplar plants into red.

## Data Availability

RNA-Seq raw data from six samples were deposited in the National Center for Biotechnology. Information (NCBI) under the accession number PRJNA822547.
