# Supplementary material for: Integrated Transcriptome and Metabolome Analyses Reveal the Anthocyanin Biosynthesis Pathway in AmRosea1 Overexpression 84K Poplar
Source: Front Bioeng Biotechnol. 2022 Jun 6;10:911701. doi: 10.3389/fbioe.2022.911701 (PMC9207281; doi:10.3389/fbioe.2022.911701)
Supplement: Supplementary file 1 [file DataSheet1.zip › Supplement material/Supplementary_Material.pdf]

Supplementary Material

1 Supplementary Figures and Tables

1.1 Supplementary Figures

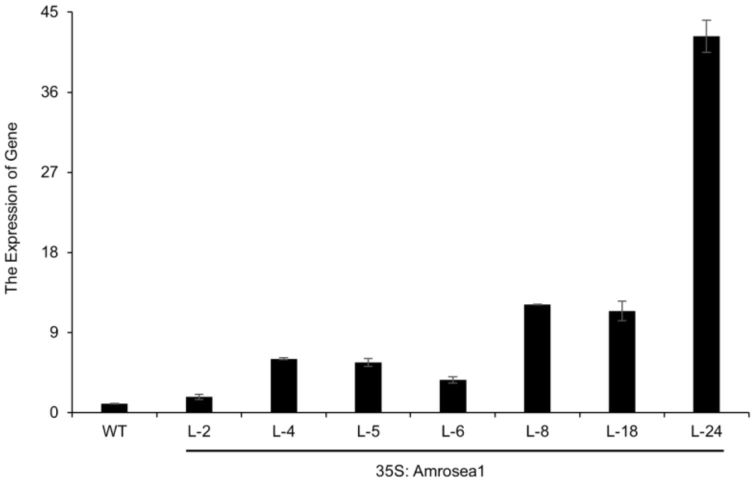

Supplementary Figure S1. Expression analysis of *AmRosea1* in wild type and transgenic lines.

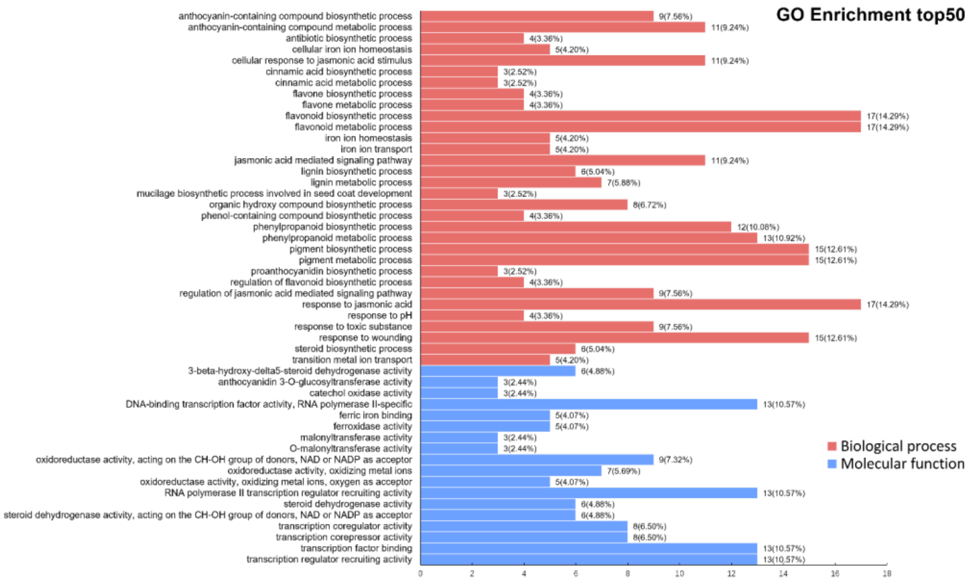

Supplementary Figure S2. Go classification of DEGs in WT vs. AM. The abscissa represents the number of DEGs annotated to the Go terms, and the value in brackets represents the ratio of DEGs in

specific term to all annotated genes. The left ordinate is the functional annotation, and the right ordinate is the major GO categories.

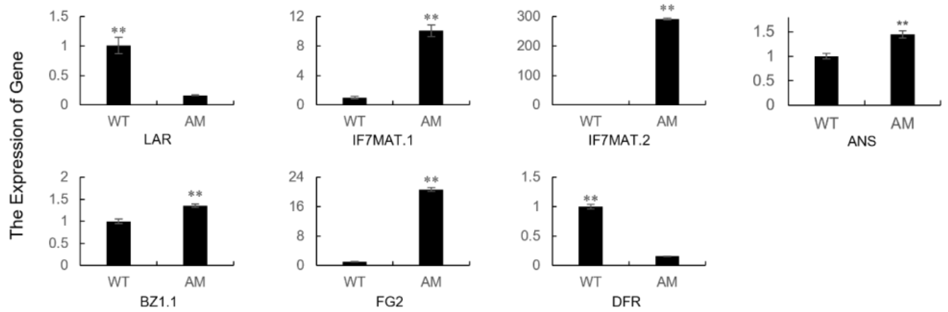

Supplementary Figure S3. The expression of genes involved in anthocyanin biosynthesis enzymes using RT-qPCR. Error bars represent the SDs from three biological replicates. Statistical differences were determined by a student's t-test (**P < 0.01).

1.2 Supplementary Table

Supplementary Table S1 RT-qPCR validation primers

Supplementary Table S2 Go classification enrichment of DEGs.

Supplementary Table S3 The sequences and annotations of key structural genes.

Supplementary Table S4 KEGG enrichment of DAMs.

删除了: Correlation analysis of DEGs and DAMs.

Supplementary Table S5 Correlation analysis of DEGs and DAMs.

删除了: KEGG enrichment of DAMs.

Supplementary Table S6 Sequence alignment rate between *AmRosea1* and *PagMYB113*.
