# Supplementary figures and images for: Integrated Transcriptome and Metabolome Analyses Reveal the Anthocyanin Biosynthesis Pathway in AmRosea1 Overexpression 84K Poplar
Source: Front Bioeng Biotechnol. 2022 Jun 6;10:911701. doi: 10.3389/fbioe.2022.911701 (PMC9207281; doi:10.3389/fbioe.2022.911701)

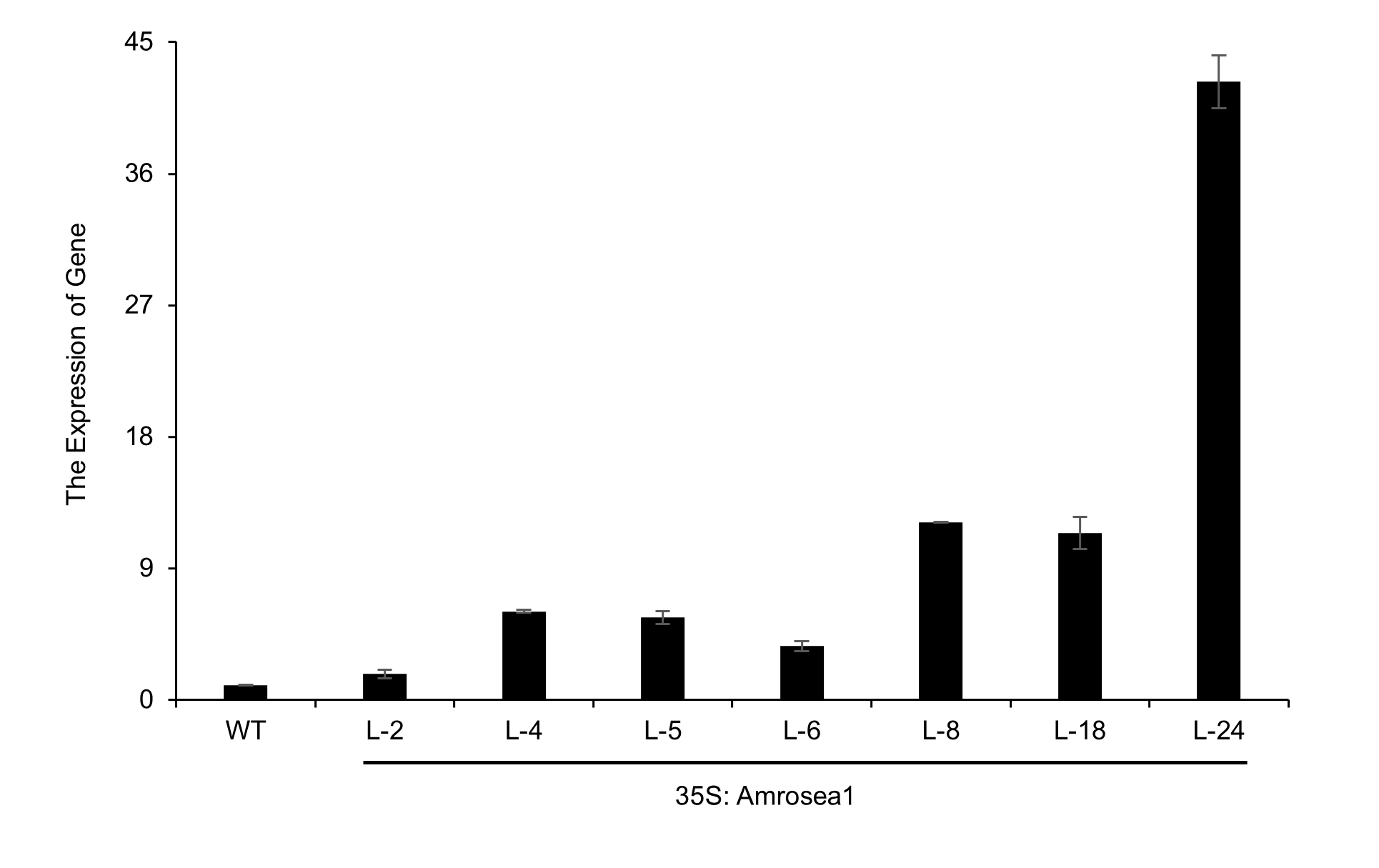

Supplement: Supplementary file 1 [file DataSheet1.zip › Supplement material/Supplement Figure S1.tif]

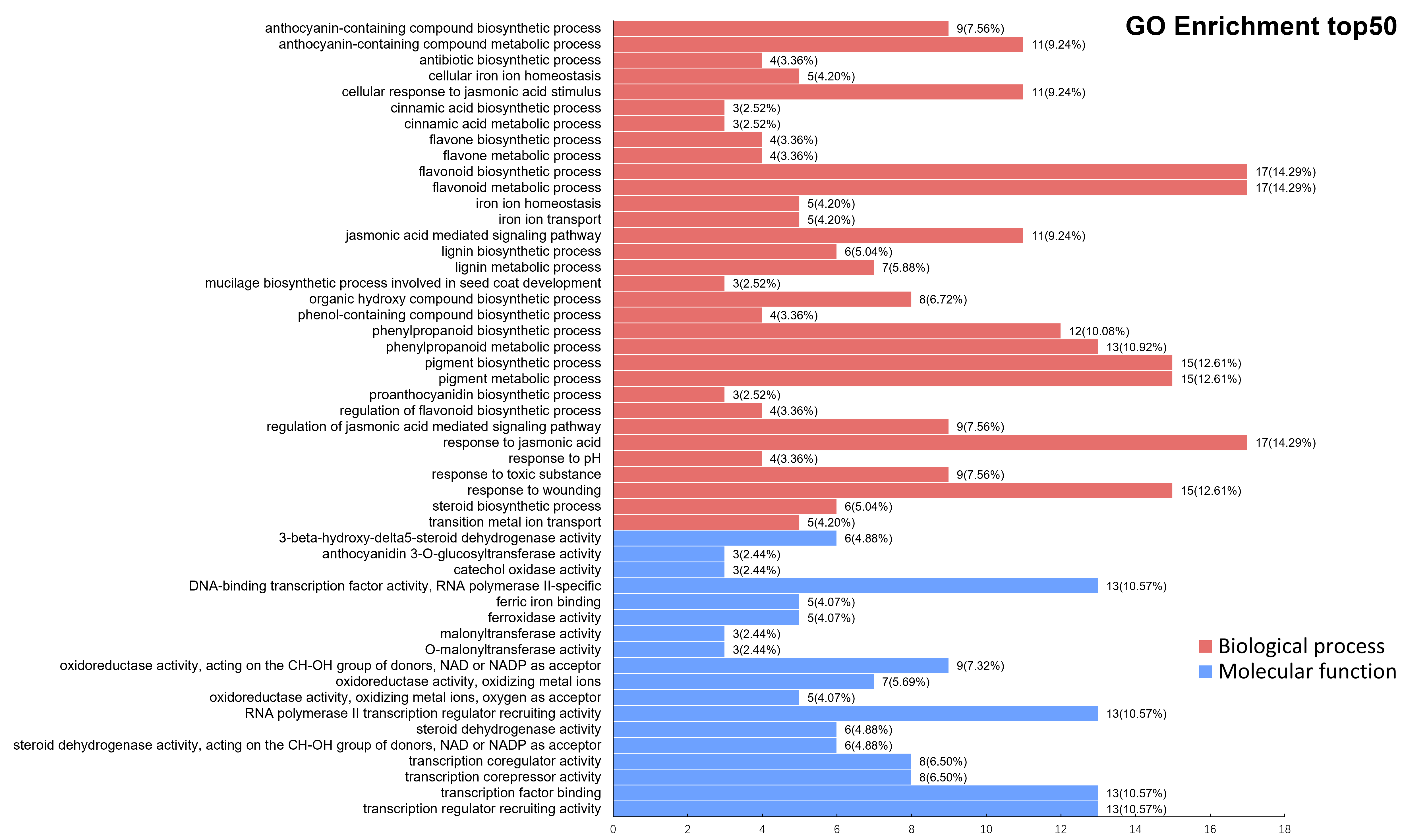

Supplement: Supplementary file 1 [file DataSheet1.zip › Supplement material/Supplement Figure S2.tif]

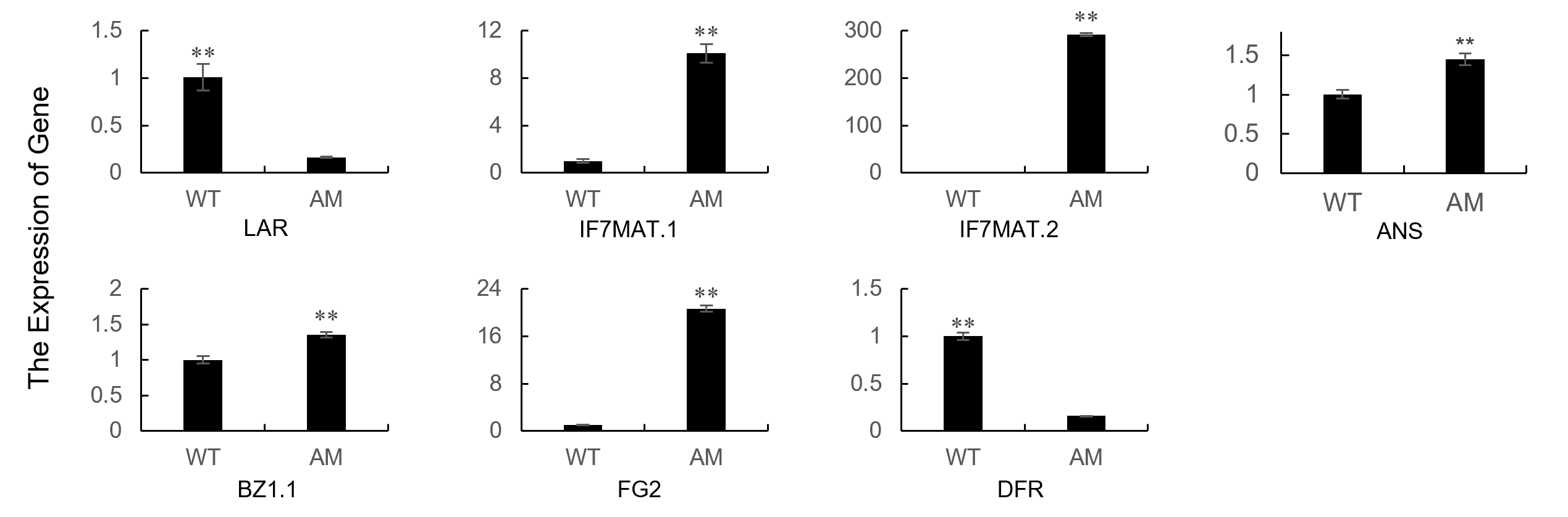

Supplement: Supplementary file 1 [file DataSheet1.zip › Supplement material/Supplement Figure S3.tif]
